# Reduced Glutamate in the Medial Prefrontal Cortex Is Associated With Emotional and Cognitive Dysregulation in People With Chronic Pain

**DOI:** 10.3389/fneur.2019.01110

**Published:** 2019-12-03

**Authors:** Brooke Naylor, Negin Hesam-Shariati, James H. McAuley, Simon Boag, Toby Newton-John, Caroline D. Rae, Sylvia M. Gustin

**Affiliations:** ^1^Neuroscience Research Australia, Sydney, NSW, Australia; ^2^School of Psychology, Macquarie University, Sydney, NSW, Australia; ^3^School of Medical Sciences, University of New South Wales, Sydney, NSW, Australia; ^4^Graduate School of Health, University of Technology Sydney, Sydney, NSW, Australia; ^5^School of Psychology, University of New South Wales, Sydney, NSW, Australia

**Keywords:** medial prefrontal cortex, chronic pain, spectroscopy, glutamate, N-acetylaspartate, harm avoidance, emotional dysregulation

## Abstract

A decrease in glutamate in the medial prefrontal cortex (mPFC) has been extensively found in animal models of chronic pain. Given that the mPFC is implicated in emotional appraisal, cognition and extinction of fear, could a potential decrease in glutamate be associated with increased pessimistic thinking, fear and worry symptoms commonly found in people with chronic pain? To clarify this question, 19 chronic pain subjects and 19 age- and gender-matched control subjects without pain underwent magnetic resonance spectroscopy. Both groups also completed the Temperament and Character, the Beck Depression and the State Anxiety Inventories to measure levels of harm avoidance, depression, and anxiety, respectively. People with chronic pain had significantly higher scores in harm avoidance, depression and anxiety compared to control subjects without pain. High levels of harm avoidance are characterized by excessive worry, pessimism, fear, doubt and fatigue. Individuals with chronic pain showed a significant decrease in mPFC glutamate levels compared to control subjects without pain. In people with chronic pain mPFC glutamate levels were significantly negatively correlated with harm avoidance scores. This means that the lower the concentration of glutamate in the mPFC, the greater the total scores of harm avoidance. High scores are associated with fearfulness, pessimism, and fatigue-proneness. We suggest that chronic pain, particularly the stress-induced release of glucocorticoids, induces changes in glutamate transmission in the mPFC, thereby influencing cognitive, and emotional processing. Thus, in people with chronic pain, regulation of fear, worry, negative thinking and fatigue is impaired.

## Introduction

Brain morphological changes are known to occur in chronic pain (1, 2). Although different types of chronic pain, e.g., nociceptive and neuropathic pain, differ in their pattern of gray matter changes (3–5), they show a substantial overlap in the medial prefrontal cortex (mPFC), where a decrease in gray matter volume has been widely demonstrated (2, 6–9). While a logical explanation for a decrease in mPFC gray matter volume is neuronal loss, there is no evidence to confirm this (10). Instead, we recently proposed that a decrease in mPFC gray matter volume reflects many changes, including vascular alterations caused by a change in metabolic activity, e.g., glutamate (10). In detail, a change in glutamate concentration effects microvasculature through neurovascular signaling and activation of pericytes which are contractile cells that line capillaries, controlling their diameter size through contraction or dilation (11).

An alteration in mPFC glutamate levels has been shown in animal models of chronic pain but has never been reported in humans with chronic pain (12–18). In animal models of chronic pain, it has been suggested that during the acute stage of pain there is an initial increase in glutamate, which is followed by a decline during the progression from acute to chronic pain (13). Thus, we would expect a decrease in mPFC glutamate in people with chronic pain.

Another important question refers to the clinical impact of such metabolic changes. Given that the mPFC is implicated in emotional appraisal (19–21), cognition (19–22) and extinction of fear (19–21), could a decrease in glutamate be associated with the increased pessimistic thinking, fear and worry symptoms commonly found in people with chronic pain? Indeed spectroscopy studies have revealed that glutamate mediates the behavioral sequelae associated with anxiety and stress (23) as well as the sequelae of pain perception itself (24, 25).

While as many as 50% of people with chronic pain suffer from anxiety and depression (26, 27), it is not known if these state factors relate to a potential decrease in mPFC glutamate. Unfortunately, no published studies have examined mPFC glutamate levels in individuals with chronic pain. As alterations in mPFC gray matter volume represent changes in mPFC glutamate levels in people with chronic pain (10), studies investigating the relationship between mPFC gray matter volume and state anxiety and depression may provide evidence about whether these state factors are related to decreased mPFC glutamate in chronic pain sufferers.

Decreased mPFC gray matter volume has been reported in anxiety disorders (28–30) and depression (31–34). To our knowledge, there are two published studies which directly link the decrease in mPFC gray matter volume to state anxiety and depression symptoms in people with chronic pain (35, 36). However, five other studies found no such relationship between mPFC gray matter decrease and state anxiety and depression in chronic pain sufferers (37–41). This suggests that there may be other emotional, cognitive and behavioral factors that are linked to a decrease in mPFC gray matter volume and hence, to a potential decline in mPFC glutamate.

Evidence is accumulating that a high level of the temperament “harm avoidance” is the most distinguishing multidimensional trait of chronic pain sufferers (42–45). Elevated harm avoidance, as per Cloninger's Temperament and Character Inventory (46), comprises cognitive, emotional, and behavioral factors characterized by excessive worry, pessimism, fear, doubt, apprehension and fatigue. In the chronic pain context, high harm avoidance usually manifests as persistent, excessive fear and worry about pain (47). Harm avoidance has previously been linked directly to mPFC metabolic and neuronal activity (48–52). Hence, we suggest that a potential decrease in mPFC glutamate may be associated with high levels of harm avoidance amongst chronic pain sufferers.

We used magnetic resonance spectroscopy to determine: (1) if individuals with chronic pain show a decrease in mPFC glutamate and (2) if this decline in mPFC glutamate is associated with negative affective state factors such as depression and anxiety or multidimensional trait factors such as harm avoidance. Metabolites including glutamate, *N*-acetylaspartate, creatine, and myo-inositol were compared between chronic pain subjects and age- and gender- matched healthy controls. Both groups also completed the Beck Depression Inventory, the State-Trait Anxiety Inventory and the Temperament and Character Inventory which measure depression, anxiety and harm avoidance, respectively. We hypothesized that individuals with chronic pain would demonstrate a decrease in glutamate within the mPFC compared to age and gender matched individuals without pain. Further, we hypothesized that a decline in glutamate levels would be linked to higher levels of harm avoidance in individuals with chronic pain. That is, the higher the reports of multidimensional trait of harm avoidance, e.g., the higher the ongoing fear and worry about pain, the lower the levels of mPFC glutamate.

## Methods

### Subjects

Nineteen subjects with chronic pain (9 males; mean [± standard deviation (SD)] age 51 ± 13 years) and 19 age- and gender- matched control subjects without pain (9 males; mean [±SD] age 49 ± 14 years) were recruited for the study. Five out of 19 chronic pain sufferers were diagnosed with painful temporomandibular disorder (TMD) using the Research Diagnostic Criteria for TMD (53). Two people with chronic pain suffered from trigeminal neuropathy (TNP), one person suffered from trigeminal post-herpetic neuralgia, and one individual had atypical trigeminal neuralgia assessed by the Liverpool Criteria (54). Ten people with chronic pain suffered from neuropathic pain after spinal cord injury (SCI) as defined by the International Association for the Study of Pain SCI pain taxonomy (55) (Table 1).

**Table 1:** Chronic pain subjects characteristics.

**Subject**	**Pain disorder**	**Pain** ** type**	**Pain** ** site**	**Pain** ** duration (years)**	**Analgesic medication**	**Pain diary** ** (VAS)**	**Scan pain** ** (VAS)**
1	Trigeminal post-herpetic neuralgia	NP	Left	7.5	None	5.06	3.0
2	Myofascial pain	NNP	Bilateral	26	150 mg/day pregabalin, 3,990 mg/day paracetamol	6.36	5.3
3	Myofascial pain	NNP	Bilateral	48	None	3.97	4.3
4	Neuropathic pain after SCI	NP	Bilateral	13	None	8.77	1.4
5	Neuropathic pain after SCI	NP	Bilateral	1.3	900 mg/day pregabalin; 120 mg/day oxycodone 60 mg/day paracetamol	8.80	7.0
6	Neuropathic pain after SCI	NP	Bilateral	10.8	600 mg/day gabapentin	4.34	1.9
7	Myofascial pain	NNP	Bilateral	14	None	4.50	2.8
8	Myofascial pain	NNP	Bilateral	5.5	None	3.20	2.9
9	Myofascial pain	NNP	Bilateral	5	None	1.92	1.4
10	Trigeminal neuropathy	NP	Bilateral	9	None	3.02	6.4
11	Atypical trigeminal neuralgia	NP	left	17	None	2.60	2.6
12	Neuropathic pain after SCI	NP	Bilateral	10.5	None	5.12	6.0
13	Neuropathic pain after SCI	NP	Bilateral	10	None	0.89	0.5
14	Neuropathic pain after SCI	NP	Bilateral	4.7	600 mg/day pregabalin	3.84	1.8
15	Neuropathic pain after SCI	NP	Bilateral	36.8	None	1.63	3.1
16	Trigeminal neuropathy	NP	Bilateral	10	None	0.56	0.6
17	Neuropathic pain after SCI	NP	Bilateral	27.5	None	4.77	3.6
18	Neuropathic pain after SCI	NP	Bilateral	34.5	None	2.80	2.8
19	Neuropathic pain after SCI	NP	Bilateral	23	None	1.73	2.2
Mean (± SD)				16.5 ± 13		3.9 ± 2.3	3.1 ± 1.9

*VAS, visual analog scale; SCI, spinal cord injury; NP, neuropathic pain; NNP, non-neuropathic pain*.

TMD is mainly a nociceptive pain condition (56) affecting the temporomandibular joint and mastication muscles. TMD is primarily related to the trigeminal nerve; however, symptoms can also occur around the neck, head and ears. TMD is characterized by ongoing aching pain and tenderness (53). TNP is a neuropathic pain condition occurring in one or more branches of the trigeminal nerve. It features continuous or long periods of background aching and burning pain with episodic sharp stabbing pain (54). Trigeminal post-herpetic neuralgia is a unilateral neuropathic pain syndrome characterized by ongoing deep aching or burning pain occurring in one or more branches of the trigeminal nerve, caused by herpes zoster (57, 58). Atypical trigeminal neuralgia is a unilateral neuropathic pain condition of a branch or branches of the trigeminal nerve, featuring constant, or long periods of mild, background burning pain with abrupt onset sharp, stabbing pain (54). All SCI subjects had complete thoracic injury with continuous shooting or burning pain in the area of sensory loss which was minimum three segments below the neurological level of injury. The International standards for neurological classification of spinal cord injury (ISNCSCI) examination (59) was used to assess the extent of spinal cord damage. Specifically, we determined the most caudal level of the spinal cord with normal sensory and motor function on both sides of the body (neurological level of injury). When there was no sensory or motor function in the sacral segments, we specified the injury as complete. The Human Research Ethics Committees of the University of New South Wales and University of Sydney approved the research and all subjects gave their informed written consent in accordance with the Declaration of Helsinki.

### Psychometric Measures

During the MRI scanning session, subjects rated their present pain intensity (Table 1). A pain diary was also completed to assess participant pain intensity during the week before the scanning session. Subjects rated their pain three times daily by making a vertical pen stroke on a 10 cm horizontal line (0 cm reflecting no pain to 10 cm reflecting maximum pain imaginable). The values were averaged to reflect the subject's chronic pain intensity. Each participant also completed the revised Temperament and Character Inventory (TCI-R) (46) to assess their level of harm avoidance. The TCI-R comprises 240 items and measures four temperament traits (Novelty Seeking, Harm Avoidance, Reward Dependence, and Persistence) and three character traits (Self-Directeness, Cooperativeness, and Self-Transcendence). Percentile scores were determined from the raw scores for harm avoidance using the following ranges: 84–100%, very high; 67–83.3%, high; 34–66.7%, average; 17–33%, low; 0–16.7%, very low. The State Anxiety Inventory (32, 60) was also completed by each participant. This measure has 20 items with scores ranging from 20 to 80. A cut-off score of 39–40 has been suggested to detect clinically significant symptoms for state anxiety (61). State anxiety reflects a temporary condition characterized by autonomic nervous system activation and feelings of fear, nervousness and tension in response to a perceived threat. Moreover, to assess depressive symptoms, the Beck Depression Inventory (62) was completed by each participant. This is a valid and reliable measure of depression in chronic pain patients (63), with scores ranging from 0–63. The standard cut-off scores are as follows, 0–9 indicates minimal depression, 10–18 indicates mild depression, 19–29 indicates moderate depression, and 30–63 indicates severe depression (64). All psychometric variables were continuous in nature.

### Proton Magnetic Resonance Spectroscopy (^1^H-MRS) Acquisition

Subjects lay supine head first on the bed of a 3T MRI scanner (Achieva TX Philips Healthcare, Best, Netherlands) with their head immobilized in a tight-fitting 32 channel head coil. One high resolution T1-weighted volumetric image covering the whole brain was acquired for each participant (turbo field echo; echo time = 2.5 ms, repetition time = 5,600 ms, flip angle = 8°, voxel size = 0.8 mm^3^). For voxel positioning, we used multi-planar (axial, sagittal, coronal) reconstructions. In the right mPFC (65), a voxel (20 × 30 × 30 mm^3^) was placed to collect proton magnetic spectra (**Figure 2A**) using the PRESS sequence (TR: 2,000 ms, TE: 32 ms, 1,024 acquisition points, bandwidth of 2 kHz, 64 averages, water suppression technique “excitation”). From the same voxel position, we also collected an unsuppressed water spectrum with 8 averages. All voxel placements were positioned based on anatomical and histological features. This was done by reference to BioImage Suite by Yale University (https://bioimagesuiteweb.github.io/webapp/) in the MNI2TAL application, against a 1.5T high resolution (1 mm) brain. Further to this, the atlas of the Human Brain (65) was used to verify final placement of the voxel to ensure that the target voxel was placed within the mPFC and not within the anterior cingulate, orbitofrontal, or dorsolateral prefrontal cortex. The voxel was targeted to land on the Superior Frontal Gyrus, Medial Part or the Superior Frontopolar Gyrus, both well within the mPFC.

### ^1^H-MRS Analysis

Java-based magnetic resonance user's interface (jMRUI 4.1, European Union project) was used for the analysis of the MRS data in the time domain. First, the Hankel Lanczos Singular Value Decomposition algorithm was employed to remove the dominant water resonance. QUEST was then used to quantify N-acetylaspartate (NAA), creatine (Cr), glutamate (Glu), and myo-inositol (MI) resonances using a 32 ms TE metabolite basis set including NAA, Cr, Glu, MI, glutamine, and glycerophosphorylcholine generated using the NMR-SCOPE tool in jMRUI using coupling constant and chemical shift information from Gasparovic et al. (66). Ratios were calculated for NAA, Cr, Glu, and MI relative to H_2_O which was measured from the unsuppressed water reference spectrum. H_2_O ratios have been used because H_2_O provides a more robust reference compared to Cr which can vary across the brain even in non-disease states and particularly in healthy aging (67–70).

### Spectral Quality Assessment and Voxel Tissue Content Analysis

Variances were calculated from the peak areas and the standard deviations of the fit for each metabolite in each subject to assess the goodness of fit. Average linewidths and signal to noise ratios (SNR) were also examined. Signal-to-noise ratios were measured using the peak amplitudes of NAA in the water suppressed spectrum compared to the peak amplitude of the noise from a signal free section of the spectrum around 10 ppm in each subject. Spectroscopy region of interests (ROIs) were assessed for relative fraction of cerebrospinal fluid, gray and white matter using a tool for partial volume estimation of Philips data (66).

### Statistical Methods

All data was analyzed using SPSS statistical software (version 25). The Shapiro-Wilk test was used to assess the normal distribution of all data. Data identified as parametric was analyzed with two-tailed independent *t*-tests to assess for between group comparisons. Two-tailed Pearson correlations were also used to determine any significant correlations between parametric data variables. A stepwise sequential regression model [including Akaike's Information Criterion with small-sample correction (AICc) fit and forward selection with harm avoidance as the outcome and NAA and glutamate as predictors] was used to assess the influence of NAA and glutamate on harm avoidance. During the study, a significance level of *p* < 0.05 was employed, with the Bonferroni-Holm correction used for multiple comparisons and respective cumulative α error.

## Results

On average (mean ± SD), chronic pain subjects had on-going pain intensity of 3.9 ± 2.3 (diary pain), pain intensity during scanning of 3.1 ± 1.9 (scan pain), and an average pain duration of 16.5 ± 13 years. Table 1 shows the individual and mean chronic pain participant characteristics. Seventeen out of 19 chronic pain subjects had bilateral pain and two had left-sided pain. Fourteen out of 19 chronic pain subjects had neuropathic pain and five had non-neuropathic pain (myofascial pain). Ten out of 19 chronic pain sufferers had neuropathic pain after SCI and nine had orofacial pain (Table 1). There was no significant difference in age for chronic pain subjects compared to controls without pain (mean [±SD] age: chronic pain subjects: 51 ± 13; control subjects without pain: 49 ± 14; *p* = 0.68, computed test statistic (*t*) = 0.43, degrees of freedom (*df*) = 36).

### Psychometric Measures

**Figure 1:**
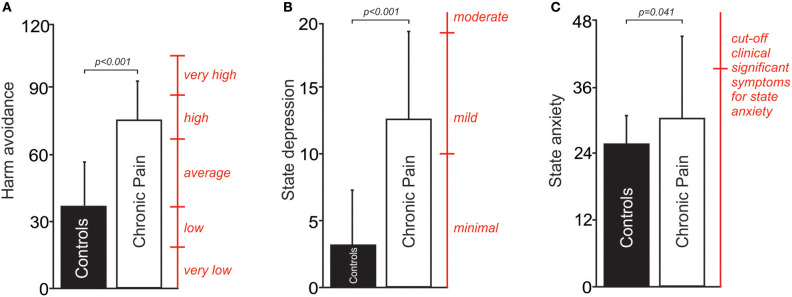
**(A)** A plot of mean (±SD) levels of harm avoidance in people with chronic pain and pain-free controls. Percentile scores (red) derived from a standard community sample of 300 normal adult individuals; 84–100%, very high; 67–83.3%, high; 34–66.7%, average; 17–33%, low; 0–16.7%, very low (71). **(B)** A plot of mean (±SD) depression scores in people with chronic pain and pain-free controls. Cut-off scores (red) of 0–9 indicate minimal depression, 10–18 indicate mild depression, and 19–29 indicate moderate depression (64). **(C)** A plot of mean (±SD) anxiety scores in people with chronic pain and pain-free controls. A cut-off score (red) of 39–40 has been suggested to detect clinically significant symptoms for state anxiety (61).

Chronic pain subjects had significantly higher scores in harm avoidance compared to the age and gender matched control group without pain (mean [±SD] harm avoidance: chronic pain subjects: 74 ± 22; control subjects without pain: 34 ± 23; *p* < 0.001, *t* = 5.32, *df* = 36; Figure 1A). In addition, chronic pain subjects had high values in harm avoidance when compared to a standard community sample of 300 normal adult individuals (71). In contrast, the control group had average levels of harm avoidance compared to the standard community sample (71). There was no significant difference in harm avoidance levels between subjects with neuropathic pain (*n* = 14) and subjects with non-neuropathic pain (myofascial pain, *n* = 5) (mean [±SD] harm avoidance: neuropathic pain subjects: 69 ± 23; non-neuropathic pain subjects: 86 ± 17; *p* = 0.14, *t* = −1.77, *df* = 9).

Chronic pain subjects had significantly higher depression scores when compared to the age and gender matched control group without pain (mean [±SD] depression scores: chronic pain subjects: 12 ± 7; control subjects without pain: 3 ± 4; *p* < 0.001, *t* = 4.50, *df* = 26; Figure 1B). There was no significant difference in depression scores between subjects with neuropathic pain (*n* = 14) and subjects with non-neuropathic pain (myofascial pain, *n* = 5) (mean [±SD] depression scores: neuropathic pain subjects: 11 ± 7; non-neuropathic pain subjects: 14 ± 8; *p* = 0.33, *t* = −1.01, *df* = 17).

Finally, chronic pain subjects had significantly higher scores in state anxiety when compared to the age and gender matched control group without pain (mean [±SD] state anxiety score: chronic pain subjects: 31 ± 12; control subjects without pain: 25 ± 5; *p* = 0.041, *t* = 2.20, *df* = 36; Figure 1C). There was no significant difference in anxiety scores between subjects with neuropathic pain (*n* = 14) and subjects with non-neuropathic pain (myofascial pain, *n* = 5) (mean [±SD] anxiety scores: neuropathic pain subjects: 28 ± 8; non-neuropathic pain subjects: 39 ± 16; *p* = 0.17, *t* = −1.60, *df* = 5).

### Differences in mPFC Resonance Levels Between Chronic Pain and Control Subjects

**Figure 2:**
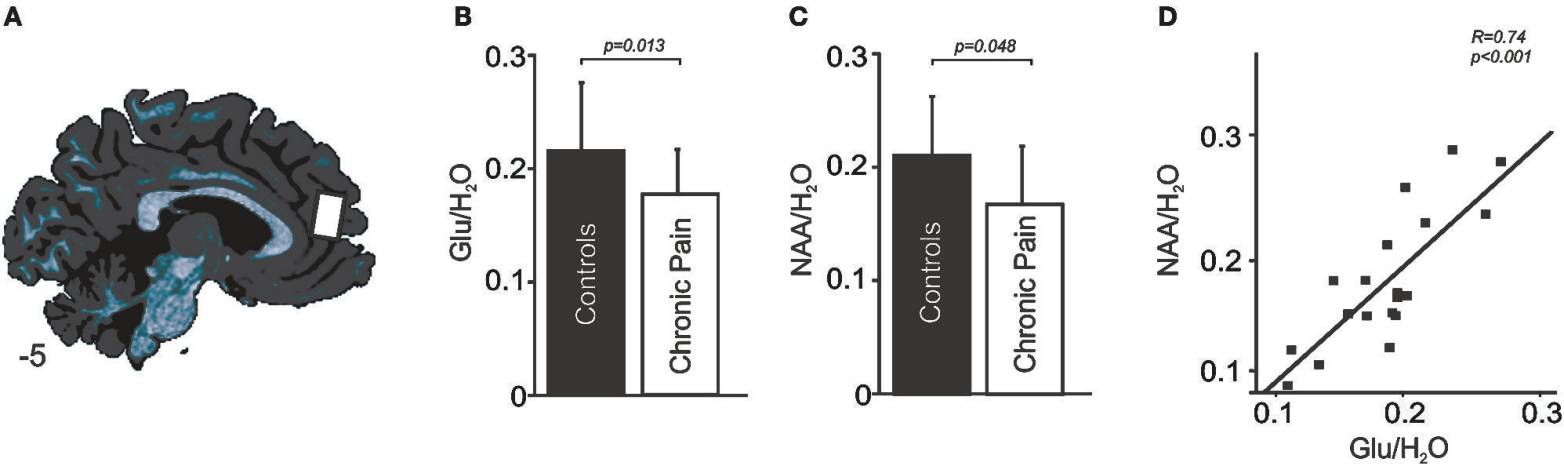
**(A)** Sagittal slice showing location from which proton spectroscopy was performed in the right medial prefrontal cortex in people with chronic pain and pain-free controls. Slice location in Montreal Neurological Institute space is indicated at the lower left of the image. **(B)** A plot of mean (±SD) Glu/ H_2_O ratios in the medial prefrontal cortex in people with chronic pain and pain-free controls. **(C)** A plot of mean (±SD) NAA/H_2_O ratios in the medial prefrontal cortex in people with chronic pain and pain-free controls. **(D)** A plot of Glu/ H_2_O ratios in people with chronic pain against NAA/ H_2_O ratios in the medial prefrontal cortex.

The mPFC voxel from which ^1^H-MRS spectra was acquired is shown in Figure 2A. Chronic pain subjects had significantly lower mPFC glutamate levels compared with age and gender matched healthy control subjects (mean [±SD] Glu/H2O ratio: chronic pain subjects: 0.18 ± 0.04; control subjects without pain: 0.22 ± 0.05; *p* = 0.013, *t* = −2.65, *df* = 35; Figure 2B). There was no significant difference in mPFC glutamate levels between subjects with neuropathic pain (*n* = 14) and subjects with non-neuropathic pain (myofascial pain, *n* = 5) (mean [±SD] Glu/H_2_O ratio: neuropathic pain subjects: 0.18 ± 0.04; non-neuropathic pain subjects: 0.19 ± 0.03; *p* = 0.46, *t* = −0.75, *df* = 17).

Chronic pain subjects had significantly lower mPFC NAA levels compared with control subjects without pain (mean [±SD] NAA/H_2_O ratio: chronic pain subjects: 0.17 ± 0.05; control subjects without pain: 0.21 ± 0.05; *p* = 0.048, *t* = −2.05, *df* = 36; Figure 2C). There was no significant difference in mPFC NAA levels between subjects with neuropathic pain (*n* = 14) and subjects with non-neuropathic pain (myofascial pain, *n* = 5) (mean [±SD] NAA/H2O ratio: neuropathic pain subjects: 0.17 ± 0.06; non-neuropathic pain subjects: 0.17 ± 0.04; *p* = 0.82, *t* = 0.24, *df* = 17).

Finally, chronic pain subjects had similar mPFC MI and Cr levels compared with control subjects (mean [±SD] MI/H_2_O ratio: chronic pain subjects: 0.20 ± 0.04; control subjects without pain: 0.20 ± 0.08; *p* = 0.61, *t* = −0.52, *df* = 36; mean [±SD] Cr/H_2_O ratio: chronic pain subjects: 0.22 ± 0.04; control subjects without pain: 0.24 ± 0.06; *p* = 0.36, *t* = −0.92, *df* = 36).

### Relationship Between mPFC Resonances and Psychometric Measures

**Figure 3:**
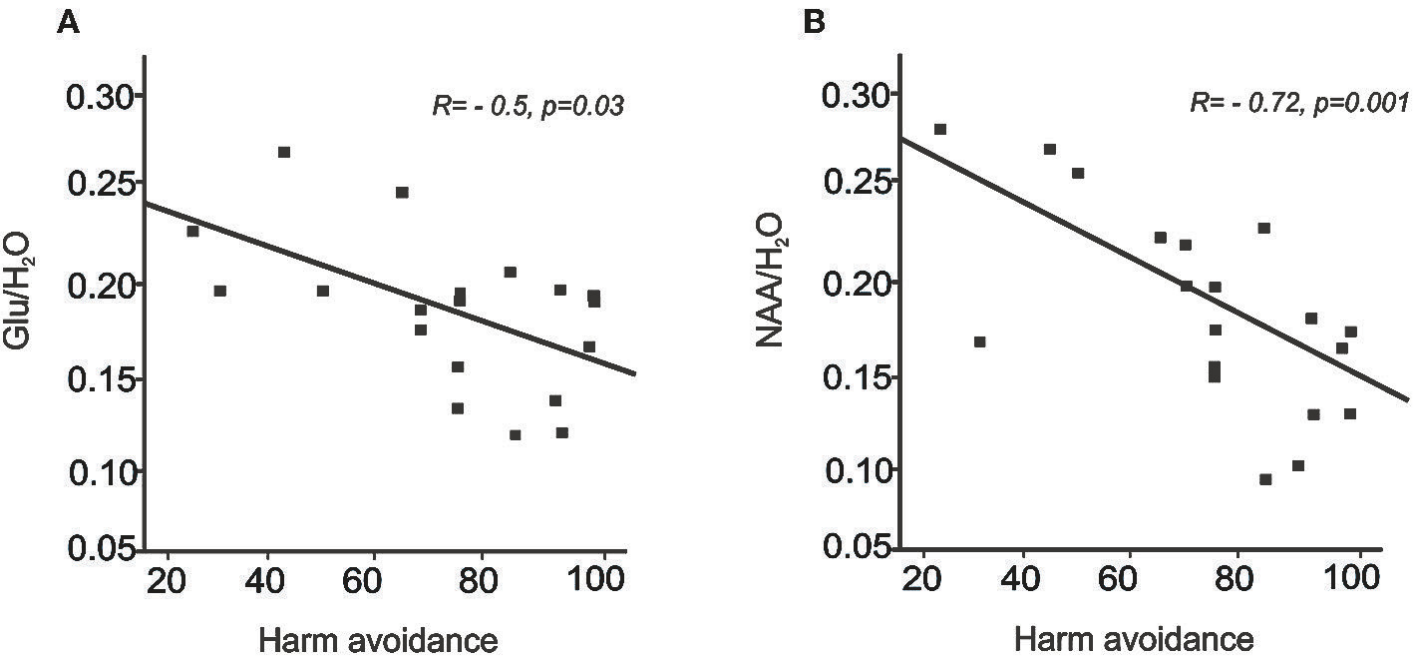
**(A)** A plot of Glu/ H_2_O ratios in the medial prefrontal cortex in people with chronic pain against levels of harm avoidance. **(B)** A plot of NAA/H_2_O ratios in the medial prefrontal cortex in people with chronic pain against levels of harm avoidance.

In the chronic pain group, Glu/H_2_O ratios were negatively correlated to levels of harm avoidance (*R* = −0.5, *p* = 0.03), that is the greater the reduction in medial prefrontal cortex glutamate levels, the higher the levels in harm avoidance (Figure 3A). In contrast, no significant correlation between Glu/H_2_O ratios and levels of harm avoidance was found in controls without pain (*R* = −0.188, *p* = 0.46). Furthermore, in chronic pain subjects Glu/H_2_O ratios were not correlated to either diary pain (*R* = 0.03, *p* = 0.90), scan pain (*R* = 0.4, *p* = 0.87), pain duration (*R* = 0.34, *p* = 0.16), state depression (*R* = −0.20, *p* = 0.42), or state anxiety (*R* = −0.27, *p* = 0.27). In control subjects without pain, Glu/H_2_O ratios were also not correlated to either state anxiety (*R* = −0.13, *p* = 0.62) or state depression (*R* = −0.40, *p* = 0.10).

In chronic pain subjects, NAA/H_2_O ratios were negatively correlated to levels of harm avoidance (*R* = −0.7, *p* = 0.001); that is, the greater the reduction in medial prefrontal cortex NAA levels, the higher the levels of harm avoidance (Figure 3B). In contrast, no significant correlation between NAA/H_2_O ratios and levels of harm avoidance was found in controls without pain (*R* = −0.01, *p* = 0.96). Furthermore, in chronic pain subjects NAA/H_2_O ratios were not correlated to either diary pain (*R* = 0.09, *p* = 0.18), scan pain (*R* = −1.25, *p* = 0.61), pain duration (*R* = 0.44, *p* = 0.06), state depression (*R* = 0.09, *p* = 0.72), or state anxiety (*R* = −0.04, *p* = 0.88). In control subjects without pain, NAA/H_2_O ratios were also not correlated to either state anxiety (*R* = 0.42, *p* = 0.07) or state depression (*R* = 0.22, *p* = 0.36).

In chronic pain subjects, MI/H_2_O ratios were not correlated to levels of harm avoidance (*R* = 0.48, *p* = 0.85), diary pain (*R* = −0.07, *p* = 0.77), scan pain (*R* = −0.10, *p* = 0.65), pain duration (*R* = 0.10, *p* = 0.97), state depression (*R* = −0.13, *p* = 0.60), or state anxiety (*R* = −0.19, *p* = 0.44). In controls without pain MI/H_2_O ratios were also not correlated to either state anxiety (*R* = −0.39, *p* = 0.10), state depression (*R* = −0.02, *p* = 0.94), or harm avoidance (*R* = 0.015, *p* = 0.952).

In chronic pain subjects, Cr/H_2_O ratios were not correlated to levels of harm avoidance (*R* = −0.35, *p* = 0.14), diary pain (*R* = −0.07, *p* = 0.77), scan pain (*R* = −0.10, *p* = 0.65), pain duration (*R* = 0.35, *p* = 0.14), state depression (*R* = 0.02, *p* = 0.94), or state anxiety (*R* = −0.20, *p* = 0.41). In control subjects without pain, MI/H_2_O ratios were also not correlated to levels of harm avoidance (*R* = −0.25, *p* = 0.30), state anxiety (*R* = −0.23, *p* = 0.36), or state depression (*R* = −0.12, *p* = 0.66).

In both chronic pain subjects and control subjects, Glu/H_2_O ratios were positively correlated to NAA/H_2_O ratios (chronic pain subjects: *R* = 0.74, *p* < 0.001, Figure 2D; control subjects: *R* = 0.61, *p* = 0.008). Furthermore, in chronic pain subjects, the relationship of NAA to harm avoidance remained significant when we accounted for glutamate in a sequential regression model [*p* = 0.009, *R*^2^ = 0.49 (AICc = 165.6)]. In contrast, the relationship of glutamate to harm avoidance was no longer significant when we accounted for NAA in a sequential regression model [*p* = 0.88, *R*^2^ = 0.049 (AICc = 169.7)].

### ^1^H-MRS Partial Volume Makeup

There was no significant difference in the fraction of cerebrospinal fluid, gray, and white matter within the mPFC voxel between chronic pain and control subjects (gray matter percentage within the mPFC voxel: chronic pain subjects: 0.23 ± 0.16 mean [±SD]; control subjects: 0.24 ± 0.19 mean [±SD]; *t* = −0.07, *df* = 35, *p* = 0.94; white matter fraction within the mPFC voxel: chronic pain subjects: 0.06 ± 0.05 mean [±SD]; control subjects: 0.18 ± 0.26, *t* = −1.92 mean [±SD], *df* = 19.41, *p* = 0.07; mean [±SD]; cerebrospinal fluid fraction within the mPFC voxel: chronic pain subjects: 0.70 ± 0.15 mean [±SD]; control subjects: 0.58 ± 0.34 mean [±SD]; *t* = 1.42, *df* = 25.17, *p* = 0.17).

### Spectral Quality Assessment

According to the consensus on clinical proton MRS of the brain (72) the linewidths, SNR and variances of the metabolites were all well within acceptable limits for data quality. Line widths for all spectra were <10 Hz after automatic shimming (pencil beam auto second order option). Furthermore, there was no significant difference in each metabolite's mean variance (%) between chronic pain and control subjects [NAA variance mean ± SD: chronic pain subjects: 8.2 ± 4.8% (minimum 3.5; maximum 19.0); control subjects: 7.0 ± 2.0% (minimum 4.2; maximum 11.0); *t* = 0.97, *df* = 25, *p* = 0.34; Glu variance mean ± SD: chronic pain subjects: 15.0 ± 5.8% (minimum 3.8; maximum 19.8); control subjects: 15.4 ± 5.2% (minimum 3.4; maximum 19.9); *t* = −0.24, *df* = 35, *p* = 0.81; MI variance mean ± SD: chronic pain subjects: 7.6 ± 2.6% (minimum 3.4; maximum 12.0); control subjects: 8.1 ± 4.6% (minimum 4.2; maximum 19.4); *t* = −39, *df* = 35, *p* = 0.70; Cr variance mean ± SD: chronic pain subjects: 5.0 ± 2.0% (minimum 2.4; maximum 9.1); control subjects: 5.2 ± 2.1% (minimum 2.6; maximum 11.0); *t* = −0.37, *df* = 35, *p* = 0.72]. There was also no significant difference in mean SNR ratios between chronic pain and control subjects [SNR ratios mean ± SD: chronic pain subjects: 30 ± 14.7 (minimum 7.7; maximum 54); control subjects: 24 ± 12.1 (minimum 3.5; maximum 54); *t* = 1.39, *df* = 35, *p* = 0.17].

## Discussion

This study demonstrates that chronic pain is associated with a significant reduction in glutamate in the mPFC. Glutamate is known as the major excitatory neurotransmitter in the brain (73). Furthermore, the study revealed that people with chronic pain show a decrease in NAA, a marker of neuronal integrity (74), in the mPFC, compared to age- and gender- matched individuals without pain. Additionally, in chronic pain subjects, both metabolites glutamate and NAA were significantly negatively correlated to harm avoidance. That is, the higher the multidimensional trait of harm avoidance, e.g., the higher the ongoing fear and worry about pain, the lower the levels of mPFC glutamate and NAA. In contrast, no significant relationship between either metabolite and harm avoidance was found in healthy subjects. Moreover, no significant relationship was found between either metabolite and state anxiety and depression in healthy or chronic pain subjects. Finally, we found a significant positive relationship between glutamate and NAA.

NAA is well-known to be a marker of both neuronal loss and mitochondrial activity (69). In our study, we argue that the decline in mPFC NAA represents a decrease in mitochondrial activity rather than neuronal loss because (1) we also found a reduction in mPFC glutamate which declines with decreased mitochondrial activity in a linear manner (69, 75, 76) and (2) we found a significant positive correlation between NAA and glutamate. In line with this argument, evidence is arising that a reduction in NAA is related to glutamate dysfunction (77, 78). The positive association between NAA and glutamate may reflect NAA's involvement in facilitating energy metabolism in neuronal mitochondria from glutamate (78). Furthermore, multimodal brain imaging studies point away from neuronal loss as the likely explanation for mPFC gray matter volume decline in people with chronic pain (79). Indeed, we recently suggested that in individuals with chronic pain, a decrease in mPFC gray matter volume does not represent neuronal loss but rather a dysregulation in glutamate metabolism (10).

Our study revealed no significant difference between the fraction of gray matter within the mPFC voxel between control and chronic pain subjects. This could be explained by the variance (>30%) in the amount of gray matter across the age range from 23 to 68 studied here, being greater than the reported changes in mPFC gray matter [12%, (80)] and whole brain gray matter volume [5.4%, (81)] in chronic pain subjects compared to healthy control subjects. Hence, the chances of finding a difference in gray matter within the mPFC box between both groups studied here is minimal.

The mPFC is rich in glutamatergic cells and innervation (82, 83). Both increased (12, 14, 16, 18) and reduced mPFC glutamate (13, 15, 17) have been shown in the animal model of chronic pain. Furthermore, reduced glutamate has been found in the anterior cingulate cortex in individuals with both acute and chronic pain (25, 84–86). Guida et al. suggested that an initial glutamate increase during the acute phase of pain is followed by a decline during its progression to chronicity (13). This suggestion aligns with findings, as we presented here, of a decrease in mPFC glutamate in people with long-term, chronic pain. The interesting question to ask is which cellular mechanisms underlie this decrease in mPFC glutamate in people with chronic pain. It is well-known that the mPFC is specifically vulnerable to the effects of stress (87–89). It is also well-established that acute pain can be perceived as an acute stressor which can evoke a physiological stress response, e.g., release of glucocorticoids such as cortisol (73, 90). In acute pain, the stress-induced release of glucocorticoids rapidly increases glutamate release in the mPFC (91, 92). In particular, acute stress induces a rise of readily releasable glutamate vesicles in the mPFC (73). In contrast, the effects of chronic stress, and hence chronic pain, on glutamate release are mostly unknown (73). It is thought that chronic pain results in sustained mPFC glial cell changes (93–95), which alter glutamate neurotransmission in the mPFC (73, 96, 97). In particular, high levels of glucocorticoids result in glia activation (97) leading to the production of cytokines, which downregulate glutamate function (98). The downregulation of mPFC glutamate function may reflect a decrease in mPFC glutamate, as measured by magnetic resonance spectroscopy in our study.

The sequential regression model used in our study (including harm avoidance as outcome and NAA and glutamate as predictors) revealed that NAA may be key in the relationship between glutamate and harm avoidance. This is not surprising as it has been proposed that NAA may act as a reservoir of glutamate (77). Particularly, Clark et al. suggested that NAA is converted to aspartate in oligodendrocytes, which can then be converted to glutamate through the TCA cycle with an energetically favorable set of reactions (77). Thus, NAA in neuronal tissue may serve as a large reservoir for refilling glutamate (77), and hence may be key in shaping the relationship between glutamate and harm avoidance in times of stress.

Another important question refers to the clinical impact of a decrease in mPFC metabolic activity. In individuals with chronic pain, both glutamate and NAA were significantly negatively correlated with harm avoidance, but not with state depression and anxiety scores. As anxiety and depression scores were not correlated to glutamate and NAA levels, it seems that the decrease in glutamate and NAA is associated with different constructs than state anxiety and depression in individuals with chronic pain. The mPFC is implicated in complex cognitive functions such as learning and memory (21, 99), decision making (22), executive control (100), and emotional processing (19) such as extinction of fear (20). Given this, it is conceivable that changes in concentration of these metabolites may alter more complex multidimensional trait factors.

Harm avoidance is a multidimensional trait comprising cognitive, emotional, and behavioral characteristics and aligns with the psychological complexity of chronic pain presentations (47, 101). Harm avoidance refers to the psychological correlates of fear and worry, but it also refers to other symptom characteristics which compound adjustment to chronic pain such as pessimistic thinking, chronic tiredness, fatigue-proneness, and sensitivity to criticism and punishment (46).

Both rodent and human studies have revealed that the mPFC is critical in fear conditioning (20, 102–105). Chronic pain sufferers habitually experience pain as a threat from which they need to escape and at any time possibly avoid (106). This experience results in conditioned fear where fear constitutes an adaptive response to immediate threat (107). Glutamate mediates conditioned fear responses which can lead to maladaptive behavior (23), which in turn can manifest in fatigue (108), mood disorders (109, 110), and anxiety (20). This aligns with the current study, which reveals that chronic pain is associated with reduced mPFC glutamate content, which in turn is significantly correlated to fearfulness, worry, pessimism, fatigue-proneness and sensitivity to criticism and punishment. That is, the more mPFC glutamate decreases the more people with chronic pain tend to anticipate pain with fearful and pessimistic thoughts, resulting in maladaptive behavior such as fatigue-proneness (111) and avoidance of feared activities (47, 101). We suggest that the mPFC has lost its ability to extinguish fears and worries due to the decrease in mPFC glutamate concentration, resulting in an ongoing tendency toward fearfulness, pessimism, and fatigue-proneness. Indeed, alterations in mPFC glutamate concentrations directly affect mPFC glutamateric projections to the periaqueductal gray (PAG) (112). These projections are known to be critical in the cortical modulation of pain and fear responses mediated by the PAG (113, 114). Thus, changes in mPFC glutamateric projections to the PAG result in persistent fear and worry.

Furthermore, in individuals with chronic pain, mPFC NAA concentration was significantly negatively correlated with levels of harm avoidance. As described above, we argue that NAA can be seen as a marker of metabolic activity in our study. Therefore, this negative association supports our result that the lower mPFC metabolic activity, e.g., glutamate and NAA concentration, the higher the multidimensional trait of harm avoidance, e.g., more fear, worry, and pessimistic thinking.

In healthy controls, harm avoidance scores were not correlated with mPFC glutamate and NAA concentration. The lack of relationships may be because of an insufficient range in harm avoidance scores in the healthy control group. In contrast, it may suggest important causal effects. If harm avoidance was itself associated with levels of mPFC glutamate and NAA, then a similar relationship between these metabolites and harm avoidance would have occurred in both control and chronic pain subjects. The unique relationship between mPFC glutamate, NAA and harm avoidance in only chronic pain subjects suggests that any association between mPFC glutamate, NAA and harm avoidance likely appears after the development of chronic pain. Notably, we found the same pattern in a previous study—trait depression scores were only correlated to changes in gray matter volume in the thalamus, the cingulate, the dorsolateral prefrontal and hippocampal cortices in chronic pain subjects, but not in age and gender matched healthy controls (115).

Thus, with the onset of chronic pain, changes in mPFC metabolic activity may be induced. In particular, chronic pain may result in mPFC glia activation (93–95). This may lead to the production of cytokines, which downregulate glutamate function in the mPFC (73, 96, 97) that in turn may negatively affect chronic pain suffers' behavioral and emotional traits. For example, pain sufferers show the following negative behavioral and emotional characteristics: fearfulness, pessimism and fatigue-proneness. Indeed, we recently revealed that subtle alterations in prefrontal brain structure and metabolism can change an individual's personality trait in chronic pain (116).

## Limitations

Our sample size was small and therefore our results should be validated in a larger sample. We are confident that our results accurately reflect the nature of mPFC metabolic changes associated with chronic pain as, although our subject numbers were limited, both groups of chronic pain and healthy subjects were comparable on age and gender demographics. This is particularly important as we know that the mPFC changes its structure and function across the lifespan (117). It is possible that subtle differences between dissimilar types of chronic pain may emerge in a larger sample. In our sample there was no significant difference between people with neuropathic and non-neuropathic (nociceptive) pain in mPFC glutamate and NAA concentration, as well as harm avoidance, anxiety and depression scores. Indeed, we recently demonstrated that high levels of harm avoidance (113), state anxiety and depression (118) are independent of chronic pain type, e.g., neuropathic and non-neuropathic (nociceptive). A decrease in mPFC gray matter volume which may reflect a decline in mPFC glutamate (10) has been consistently shown in various pain disorders (6). Therefore, alterations in mPFC glutamate may also be independent of chronic pain type. Notably, in our study both neuropathic and non-neuropathic groups, were not significantly different in mean age, pain duration, scan pain and diary pain. Further, it is possible that some analgesics may have an effect on mPFC metabolic activity as well as on harm avoidance, depression and anxiety levels. For example, monoaminergic-based antidepressants have been shown to affect glutamate system function (119). Further, in healthy subjects, painful stimulation during treatment with morphine has resulted in decreased Glu/Cr, MI/Cr, and NAA/Cr ratios in the anterior cingulate cortex (74). Another study in healthy subjects revealed that Glu/Cr ratio in the anterior cingulate cortex/mPFC, insula and prefrontal cortex was reduced after 5 days of taking an opioid or a serotonin and norepinephrine reuptake inhibitor (120). Acute and chronic effects of medications in the mPFC needs to be tested in a larger sample size. A larger sample size may also identify differences relating to pain phenotype. In our study, the limited sample size may also explain the lack of association between mPFC NAA concentration and pain intensity and mPFC NAA concentration and pain duration. Previous studies have revealed that NAA concentration within the anterior cingulate cortex is associated with both pain intensity and duration in individuals with chronic pain (25, 86).

Finally, it is important to acknowledge that at 3T, glutamate and glutamine overlap (121). The fitting algorithm used here gives an estimation of glutamate concentration that is within generally acceptable error but estimation of glutamine by this method at 3T using short-echo PRESS is problematic. Given that glutamate concentrations are far greater (up to 5x higher) than glutamine (69), the uncertainty in the glutamine estimation is of limited concern.

## Conclusion

This study reveals for the first time a significant decrease in mPFC glutamate in individuals with chronic pain. Furthermore, the decrease in glutamate is significantly negatively correlated with harm avoidance. This means that the greater the mPFC glutamate decrease, the more chronic pain sufferers show the following characteristics: fearfulness, pessimism, fatigue-proneness, and sensitivity to criticism and punishment.

We suggest that chronic pain, particularly the stress-induced release of glucocorticoids, induces changes in glutamate transmission in the mPFC, thereby influencing cognitive, and emotional processing. Thus, regulation of fear, worry, negative thinking, and fatigue is impaired.

## Data Availability Statement

The datasets for this manuscript are not publicly available because the study participants did not give consent to make the data publicly available. Requests to access the datasets should be directed to SG, s.gustin@unsw.edu.au.

## Ethics Statement

The Human Research Ethics Committees of the University of New South Wales and University of Sydney approved the research and all participants gave their informed written consent in accordance with the Declaration of Helsinki.

## Author Contributions

SG designed the study. SG and BN recruited subjects, collected, analyzed data, and wrote the manuscript. NH-S, TN-J, JM, SB, and CR provided substantial contributions to the interpretation of the findings and critically revised the manuscript. All authors provided approval for publication of the content and agree to be accountable for all aspects of the work.

### Conflict of Interest

The authors declare that the research was conducted in the absence of any commercial or financial relationships that could be construed as a potential conflict of interest.
