# Corrigendum: Reduced Glutamate in the Medial Prefrontal Cortex Is Associated With Emotional and Cognitive Dysregulation in People With Chronic Pain

**DOI:** 10.3389/fneur.2022.872851

**Published:** 2022-03-16

**Authors:** Brooke Naylor, Negin Hesam-Shariati, James H. McAuley, Simon Boag, Toby Newton-John, Caroline D. Rae, Sylvia M. Gustin

**Affiliations:** ^1^Neuroscience Research Australia, Sydney, NSW, Australia; ^2^School of Psychology, Macquarie University, Sydney, NSW, Australia; ^3^School of Medical Sciences, University of New South Wales, Sydney, NSW, Australia; ^4^Graduate School of Health, University of Technology Sydney, Sydney, NSW, Australia; ^5^School of Psychology, University of New South Wales, Sydney, NSW, Australia

**Keywords:** medial prefrontal cortex, chronic pain, spectroscopy, glutamate, N-acetylaspartate, harm avoidance, emotional dysregulation

In the original article, there was an error in the **Author Contributions**. It has been updated to align with the guidelines of the International Committee of Medical Journal Editors. A correction has been made to the **Author Contributions**.

## Author Contributions

SG designed the study. SG and BN recruited subjects, collected, analyzed data, and wrote the manuscript. NH-S, TN-J, JM, SB, and CR provided substantial contributions to the interpretation of the findings and critically revised the manuscript. All authors provided approval for publication of the content and agree to be accountable for all aspects of the work.

The authors apologize for this error and state that this does not change the scientific conclusions of the article in any way. The original article has been updated.

## Publisher's Note

All claims expressed in this article are solely those of the authors and do not necessarily represent those of their affiliated organizations, or those of the publisher, the editors and the reviewers. Any product that may be evaluated in this article, or claim that may be made by its manufacturer, is not guaranteed or endorsed by the publisher.

